# Digital Public Reporting Systems for Evaluating Health Care Quality: Systematic Review

**DOI:** 10.2196/80435

**Published:** 2026-03-18

**Authors:** Lanmei Du, Reima Suomi, Elorm Damalie

**Affiliations:** 1 Department of Management and Entrepreneurship, Turku School of Economics, University of Turku, Rehtorinpellonkatu 3, Turku, 20500, Finland, 358 417296016

**Keywords:** digital health, public reporting systems, data and information process, health literacy, quality indicators

## Abstract

**Background:**

Health care public reporting (PR) refers to making information about the quality and performance of health care providers available to the public. The primary targeted use of PR is the selection of a health care provider. Previous studies suggest that PR has improved health care quality; however, the overall adoption rate of PR systems remains low. Misalignment between PR information and users’ actual needs can explain this gap.

**Objective:**

This study conducts a systematic literature review of PR systems in health care, aiming to explore how data encoding and presentation influence user utilization, with particular attention to the impact of data inconsistencies and inaccuracies on actual use.

**Methods:**

A literature search was conducted on 5 electronic databases (Web of Science, CINAHL, Embase, PsycINFO, and PubMed), focusing on studies providing information in health care from an individual perspective. Subsequently, the quality of the included studies was assessed using the Mixed Methods Appraisal Tool (McGill University). Finally, a total of 25 empirical studies were included in this study.

**Results:**

Among the 25 studies, 40% (10/25) investigated website presentation formats, 20% (5/25) explored dashboard-based data presentation formats in PR systems, 48% (12/25) examined data quality, and 20% (5/25) addressed user heterogeneity. Quantitative methods were used in 80% (20/25) of studies, while qualitative and mixed method designs accounted for 16% (4/25) and 4% (1/25), respectively. Findings suggest that standardized website and dashboard guidelines improve data reliability and user comprehension and that user heterogeneity mediates the effects of structural and process indicators on outcomes. Data collection was completed in February 2025, and the study was projected to be fully completed by September 2025.

**Conclusions:**

This study provides an integrated structure-process-outcome–based framework for PR in health care. By presenting data through dashboards, inconsistencies and inaccuracies in data across different web pages can be mitigated, thereby decreasing conflicting information and improving comprehension in PR systems. Based on the literature findings and identified knowledge gaps, this study also proposes future research directions for online PR.

## Introduction

### Background

Digital health refers to the development and use of digital technologies to enhance health-related knowledge and practices [1]. It is not only a technological concept but also profoundly shapes how people access health information. For instance, a 2023 report by Digitalis Medical indicates that 7% of all daily Google searches are health-related, equating to approximately 70,000 health care searches every minute [2].

The core function of public reporting (PR) systems is to assist individuals in selecting health care providers and services [3]. Improving the use of the PR system requires understanding how data are encoded and decoded in digital health. This understanding is crucial for individuals who use the PR system to proactively manage their health and engage in recommended behaviors [4].

Previous studies have shown that inconsistencies or errors in PR data can affect trust and reduce users’ confidence in the reporting system [5]. Such inconsistencies not only challenge data reliability but also influence how users understand PR information. However, empirical research on these data inconsistencies remains fragmented. For example, some studies suggest that information presentation affects users’ actual use of PR systems [6,7]. Moreover, these studies largely remain focused on information presentation formats, while research on data heterogeneity within systems remains limited, underscoring the complexity of PR data usage.

Patient activation measure (PAM) refers to an individual’s knowledge, skills, and confidence essential for managing their own health and health care [8,9]. Although patients show strong interest in accessing PR information to make informed health care decisions, the overall adoption of the PR system remains low, largely due to information presentations that do not match patients’ comprehension levels [10-12]. Therefore, exploring data generated in real-world physician-patient co-management contexts may help alleviate data communication challenges and support informed decision-making [13,14].

To address this gap, this study conducts a systematic literature review to provide an overview of current research on PR in digital health information systems. To achieve this objective, we address the following research questions: (1) How do system-level structural indicators influence process indicators, and subsequently influence outcome indicators? and (2) How do decoding errors in PR systems and user-system interactions influence users’ information use?

Accordingly, we systematically identified and reviewed 25 empirical studies on information presentation in PR in health care contexts. Drawing upon the structure-process-outcome (SPO) theoretical framework, we categorized and synthesized the structural indicators (website guidelines and dashboard guidelines), process indicators (data quality guidelines and data process criteria), and outcome indicators (user satisfaction and surgical mortality). Meanwhile, we position user heterogeneity as a mediating variable that links process indicators to outcome indicators. Specifically, variations in contextual and individual-level factors (age, gender, political affiliation, employment status, income, and area of residence) can influence the interactions of users’ engagement with information.

The remainder of this paper is structured as follows: the “Methods” section describes the methodology for planning and conducting the literature review, focusing on recent research; the “Results” section presents the findings in response to the 2 research questions; and the “Discussion” section discusses the progress and identifies challenges in current literature, concluding with recommendations for future research. Checklist 1 presents the PRISMA-P (Preferred Reporting Items for Systematic Review and Meta-Analysis Protocols) 2020 checklist which outlines recommended items to address in a systematic review protocol. Multimedia Appendix 1 contains the search strategies used in each database. Multimedia Appendix 2 shows the Mixed Methods Appraisal Tool (MMAT; McGill University) quality assessment report. Multimedia Appendix 3 contains the key characteristics of the included studies. Multimedia Appendix 4 includes additional details of the included studies.

### Theoretical Background

#### Overview

The presentation of information in public reports significantly influences user engagement with these systems [15]. To gain an understanding of individual users’ attitudes toward the PR system and how this forms health information adoption behavior, it is necessary to explore the information presented in the PR systems. While information is composed of data, the way data is structured determines how information is understood [16]. Based on this premise, this study assesses whether existing PR data align with users’ informational needs and examines how such alignment influences users’ adoption of PR systems.

#### PR and Quality Indicators

PR in health care refers to the disclosure of performance-related information about providers, organizations, or systems to the general public. Such information is typically nonanonymous, identifying specific professionals or institutions, thereby enabling direct comparison and accountability [17,18]. By disclosing the performance data, individuals (patients and nonpatients) can choose higher-performing providers and make health plan decisions [17,19].

The assessment of PR systems, rather than direct health care delivery, has increasingly drawn academic attention. Donabedian [20] addresses this issue by conceptualizing health care quality through the SPO framework. In health care, structure indicators refer to the system’s resources and organizational arrangements, and process indicators include all service delivery activities such as diagnosis, treatment, nursing, and patient education. Finally, outcome indicators reflect the resulting health effects, including patient health improvements, satisfaction, and mortality rates. By applying the SPO framework in this manner, we shift the focus from assessing health care delivery quality to evaluating how PR system structures and processes influence user outcomes.

In recent times, we have also seen that PR in health care is demanded from the perspective of environmental, social, and governance (ESG) reporting, which involves public disclosure of an organization’s performance and its impacts on environmental, social, and governance factors [21]. Currently, PR seems to be moving toward health care processes beyond basic clinical activity, such as PR on clinical trials [22]. In the era of the current artificial intelligence revolution, the impact of artificial intelligence on PR is, of course, an important topic [23,24].

Although defining and measuring quality in health care remains challenging, extensive evidence supports the essential role of the SPO framework in measuring health care quality [20,25]. Within the structural dimension, PR systems are an important component [25]. The health care system is depicted through structural indicators, which in turn influence process indicators, ultimately affecting outcome indicators [20]. PR has to inform its users of all these health care components. Previous research on process indicators in PR primarily focused on operational standardization, consistency, and service standard implementation [15,26]. However, few studies have examined the structural dimension from an individual-system interaction perspective.

At the process level, patient-physician collaboration (PPC) generates substantial user data [27]. During interactions between health care organizations and users, systems serve as supporting tools that can enhance treatment efficiency. The data generated in this process holds significant value for collection and use [28]. The fine details of PPC, however, remain a difficult item to document in PR.

Existing research has paid insufficient attention to patient-generated data from the PPC process, particularly in the digital era, where the processes of data collection, organization, and use remain inadequate compared to the opportunities that digital data processing could offer. Such gaps may lead to unintended consequences, including users’ mistrust of PR systems.

Finally, outcome indicators include subjective or functional metrics, such as in our sample, patient satisfaction and mortality rate [29,30]. Online PR websites enable individuals to interact directly with information, whereas individuals often rely on their own knowledge to evaluate content [17]. In contrast, in offline settings, user interaction is typically mediated by health care institutions, resulting in a more indirect experience [31].

Previous research indicates that attitudes formed through direct experience are more effective in predicting behavior than those formed through indirect experience [32]. However, studies examining interactions between individuals and system information in online environments in a PR context remain relatively limited. Taken together, these gaps highlight the need for a framework that integrates system information processing into the SPO dimensions of PR.

#### Sociotechnical Theory

According to Bostrom and Heinen [33], sociotechnical theory suggests that information systems should be designed so that the individual and technology operate as an integrated whole. In other words, the design of information systems should not only focus on data collection, processing, and transmission; the interaction between humans and information should also be emphasized. Within the context of PR, this principle is embodied through the interaction between PPC, user heterogeneity, and system-level demand for data reliability and standardization.

The operational goal of any PR system is to serve as a channel through which health care organizations and governments convey PR information to the public. This process enables individuals to filter health care information via the selection pathway and make health care choices suited to their needs [12]. PR information selection is both receptive and selective, and at the system stage, it primarily involves information processing.

In the context of PR, effective information processing is critical because it shapes how users understand and use performance data to make informed decisions. The information processing in PR systems involves a series of systematic steps that transform raw health data into actionable information for diverse stakeholders such as policymakers, providers, and the public [34]. On the PR website, data are symbols that represent the properties of objects and events [35].

Information consists of processed data, with the processing directed at increasing its usefulness [16]. System information processing involves integrating data from multiple sources into a central database, which is then retrieved and displayed on websites [36]. Regarding quality indicators, many individuals still experience significant analytical challenges, particularly when information is not clearly presented [37]. Thus, it is necessary for the PR system to reduce information asymmetries and empower patients, families, and payers to select high-quality providers [38].

These studies provide insights into the interaction between PR information and individuals, particularly regarding how end users use PR information. Previous research has primarily focused on issues of data inconsistency and low accuracy in PR [39]. However, previous studies have provided insufficient investigation into how data inconsistency issues can be effectively addressed within the field of information systems. Specifically, it is equally important to consider the objective quality of the data, incorporating data guidelines to address data inconsistency.

These findings underscore the critical role of data quality in shaping the adoption of PR systems. However, extant research demonstrates several notable limitations. First, the analytical depth remains constrained. Most studies focus primarily on information presentation as the primary mode of data delivery; however, they often overlook how data processing within systems fundamentally influences website presentation and user interpretation.

Second, investigations in the PR domain have predominantly adopted an awareness-to-behavior framework, emphasizing the role of awareness in guiding user actions [17]. Nevertheless, the complex interrelations between data quality and adoption remain underexplored, resulting in substantive gaps between PR systems and users’ actual attitudes. Consequently, there is a pressing need to examine online PR systems through a lens that integrates the system and individuals.

## Methods

### Overview

A systematic literature review requires clearly defined research questions, a transparent search process, data extraction, and data presentation [17,40]. Typical reviews cover a span of approximately 10 years. In this study, the time frame was set from 2016 to 2025. The rationale for selecting studies after 2015 is that, from this year onward, PR increasingly transitioned to internet-based platforms. The system reached its peak in July 2015, but then declined rapidly and has remained at a relatively low level since [41]. Additionally, the Hospital Consumer Assessment of Healthcare Providers and Systems star ratings system was launched, enhancing patients’ visual understanding of hospital quality information [42].

**Figure 1.**
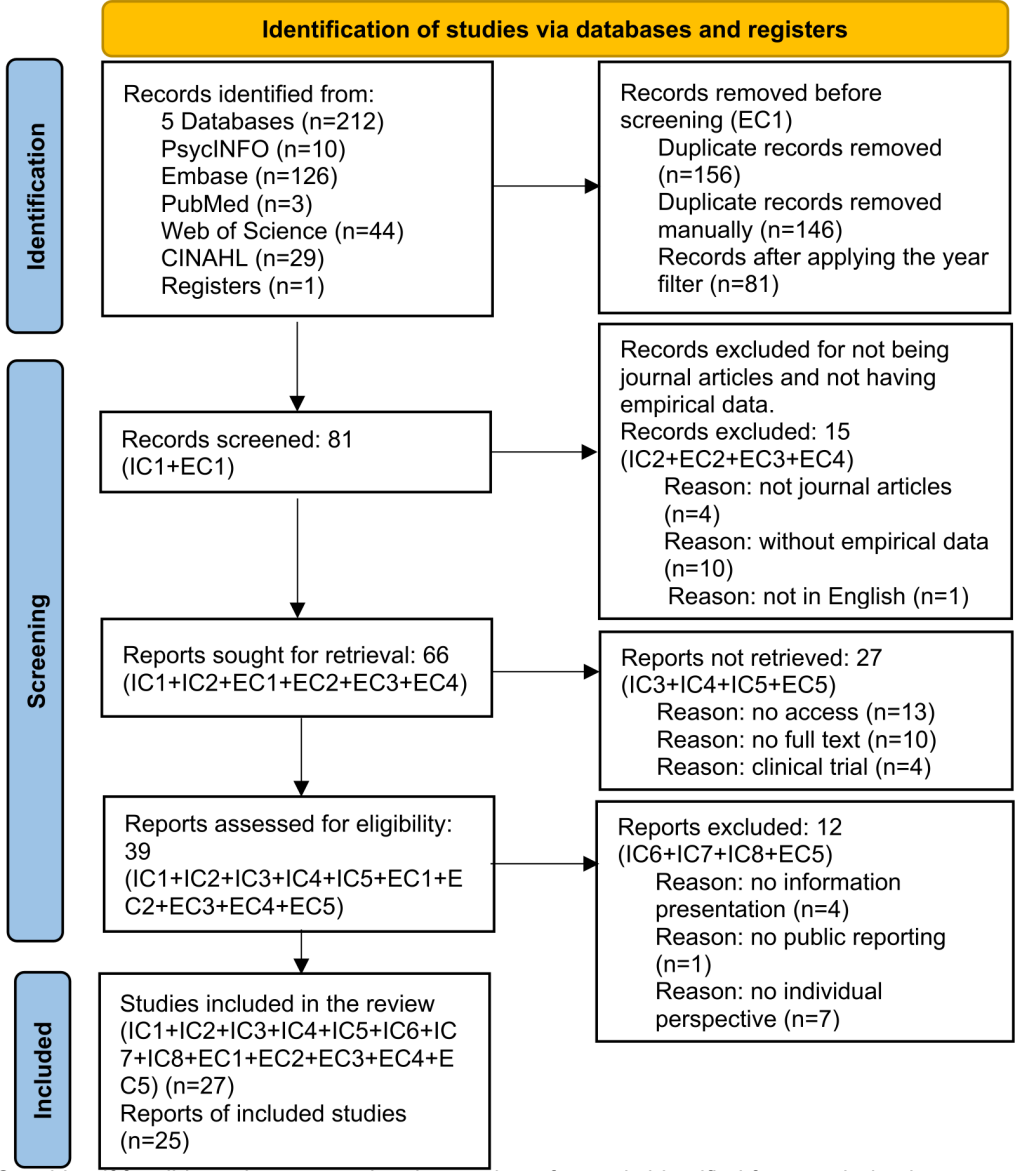
PRISMA (Preferred Reporting Items for Systematic Reviews and Meta-Analyses) flow diagram of the selection process for public reporting systems retrieved from 5 databases between 2016 and 2025. EC: exclusion criteria; IC: inclusion criteria.

We used the guidelines of Okoli and Schabram [43] and the framework proposed by Webster and Watson [44] to conduct our literature review. This review adopts a structured approach to the literature. As shown in Figure 1, the complete selection and synthesis process is summarized in the PRISMA (Preferred Reporting Items for Systematic Reviews and Meta-Analyses) flow diagram.

First, when designing the search strategy, efforts were made to capture all studies that met the inclusion criteria [45]. This research aims to explore the presentation of information in PR systems. Therefore, the search term was set to information; meanwhile, the research mainly focuses on PR, and the keyword includes “PR.” The search term is provided in Multimedia Appendix 1, and the search date was September 14, 2025.

Second, PR has evolved as an interdisciplinary field, drawing from health policy and behavioral science. Conducting a review can be helpful to fill in the gap of data inconsistency and identify future directions from the information system perspective [46]. Access to these databases was obtained via Volter (Library of the University of Turku), ensuring that the user was logged in to minimize the number of ignored studies due to access issues. The data sources were chosen from Web of Science (multidisciplinary and broad literature search), CINAHL (health and public health information systems), Embase (health and public health information systems), PsycINFO (cognitive science, decision-making, and behavioral economics), and PubMed (biomedicine literature database).

Third, using tools to assess the risk of bias in studies is an important step described in systematic review protocols, as judgments made during this process may influence the analysis and interpretation of the review results [45]. Meanwhile, applying objective and reproducible methods helps ensure the appropriateness and fairness of the study findings [43]. The quality assessment protocol covers study methodology, level of structuring, study outcomes, and research topics. After the deduplication of studies (n=146) in EndNote (Clarivate) combined with manual checking, 81 studies were included in the final scoring pool.

Among the included studies, the methodologies involved both qualitative and quantitative approaches. The scoring table referenced from Hong et al [47] is provided in Multimedia Appendix 2.

### Screening Process of the Systematic Review

In the screening process, 81 studies were initially included in the research pool. Review or descriptive studies that were not journal publications and did not contain empirical data were excluded. A total of 66 articles were retrieved, and full-text searches were conducted for these studies. During the full-text review, studies without accessible full text and clinical trial papers were excluded. Ultimately, 39 studies were included in the quality assessment.

Some of these studies examined PR systems from an organizational perspective. This study, however, focuses primarily on the individual perspective, specifically the alignment between individual users and the information presented in the PR system. Studies that explored system processes at the organizational level, which do not directly reflect the information presentation encountered by individual users, were excluded. Additionally, since this study focuses on PR systems in the health care domain, studies from non–health care fields were also excluded.

In Figure 1, abbreviated labels are used to represent inclusion criteria (IC) and exclusion criteria (EC).

The selection was conducted independently and double-checked manually. For clarity, the IC correspond to the following criteria: IC1 indicates studies published between 2016 and 2025, IC2 indicates empirical studies, IC3 indicates studies with open access, IC4 indicates studies with full text availability, IC5 indicates studies published after peer review, IC6 indicates studies focused on information presentation, IC7 indicates studies focused on the individual level, and IC8 indicates studies conducted in health care contexts. The EC corresponds to the following criteria: EC1 indicates studies published outside the 2016 to 2025 timeframe, EC2 indicates nonjournal studies, EC3 indicates nonempirical studies, EC4 indicates studies not published in English, and EC5 indicates clinical trials and other studies not focused on PR systems or individual-level information use.

After the initial full‐text screening, 27 studies were eligible for quality appraisal. The methodological quality of the included studies was assessed using the MMAT (2018 version) [47], a validated instrument specifically developed for appraising empirical studies in mixed-study systematic reviews. The MMAT evaluates 5 categories of study designs—qualitative research, randomized controlled trials, nonrandomized quantitative studies, quantitative descriptive studies, and mixed methods studies—each using 5 methodological criteria.

Following MMAT guidance, studies can be classified as low (1‐2 criteria met), moderate (3 criteria met), or high quality (4‐5 criteria met). All assessments were performed independently by the first and third authors, and discrepancies were resolved through discussion. Two studies with MMAT scores of 1/5 and 2/5 were excluded from the analysis, resulting in a final set of 25 studies for article synthesis.

### Literature Synthesis

#### Thematic and Narrative Synthesis

We conducted a thematic synthesis of the included studies to extract key findings, which were then grouped into distinct themes [45]. Within each theme, dimensional indicators were identified and presented using an integration framework to address the first research question. This approach enables the qualitative integration of evidence across studies, highlighting patterns in structure indicators, process indicators, and outcome indicators.

Given the fragmented and broad scope of research in the field of PR, a narrative synthesis approach was adopted [48]. To enhance the transparency and rigor of the synthesis, descriptive statistics were also used to summarize the number of studies within each category and their methodological characteristics.

Regarding the research questions, the theme under the SPO framework is reported quantitatively, whereas subthemes (eg, website, dashboard, and data) are explored using a narrative approach. During synthesis, studies were grouped based on the specific dimensions of the SPO framework and their relevance to the research questions. Furthermore, a subset of studies investigated how structural indicators and process indicators influence the outcome indicators. Examining findings on the same aspects allows them to extend one another, which can be viewed as a form of confirmation [48].

#### SPO Framework–Based Results Mapping

Each subtheme in the “Results” section corresponds to a specific type of data extracted from the literature: (1) structure (website and dashboard): indicators describing the system design, presentation, and functionality were collected and summarized; (2) process (data quality and data process): studies reporting on data accuracy, completeness, accessibility, and workflow processes were analyzed; (3) outcome (patient satisfaction and surgical mortality): measures were extracted from studies evaluating the effect of PR systems on users’ decision-making, satisfaction, and clinical outcomes; and (4) user heterogeneity: information on demographic characteristics, health literacy, and contextual factors influencing user interaction with PR systems was also extracted and synthesized.

#### Quality Assurance and Integration

First, we conducted an initial synthesis using thematic analysis, presenting the study results in MultimediaAppendices 3  4. Subsequently, 1 author summarized and categorized the included studies within an integrative framework. Based on this synthesis, an integrative framework was developed to organize the identified themes, which are further elaborated in the “Results” section.

## Results

### Overview

Among the 25 studies included in this review, 40% (10/25) explored PR from the perspective of information presentation formats, while 20% (5/25) of the studies focused on the dashboard in online PR. Additionally, 48% (12/25) of the studies examined PR systems from the perspective of data presentation, and 20% (5/25) addressed user heterogeneity. Finally, 12% (3/25) of the studies investigated PR from the perspective of patient satisfaction and surgical mortality.

Meanwhile, 80% (20/25) adopted quantitative methods, 16% (4/25) used qualitative designs, and 4% (1/25) used a mixed methods approach. Regarding data sources, 60% (15/25) collected secondary data (eg, web content analyses), while 40% focused on primary data through surveys, interviews, or focus groups. Data collection was conducted between February 2025 and September 2025.

### Structure

#### Website

Among the included studies, 40% (10/25) of the studies examined PR from the perspective of information presentation formats on websites. For instance, studies found that the format of information presentation on PR websites can significantly influence public understanding and trust, policymaking, and pandemic response [49].

Specifically, the findings show that standardized data guidelines help improve the quality of PR websites, including accuracy, completeness, technical elements, design and aesthetics, readability, usability, and accessibility [50]. For example, challenges in information presentation may also result from mismatches between recommended reading levels for patient education materials (typically up to sixth grade) and the actual readability of websites, which seldom meet the needs of most patients [50]. Such mismatches create a gap between the health information users need and what is actually provided. Appropriate readability facilitates comprehension, enabling better disease management and adoption of healthy lifestyles [51].

Moreover, in terms of presentation formats, we found that online ratings may serve as a valuable source of information for both consumers and policymakers [52]. It is worth noting that storytelling techniques that convey narrative trends on dashboards can help users better understand the information presented in PR [53]. Additionally, supplementing this with characteristics of professional care institutions, which are often under-represented, can enhance the comprehensiveness of PR information [54].

Furthermore, the widespread use of aggregated ratings, such as star systems, may further strengthen consumer responses [55]. Interestingly, research has shown that “breadcrumb navigation” (forward-backward topic navigation) helps maintain the continuity of information, enabling users to quickly review previously read content and efficiently locate needed information [50]. Meanwhile, studies indicate that participants accessing “drill-down scores” tend to engage more deeply with the provided data (manifested as increased interactions on the website), but their impact on physician selection is lower than that of participants accessing “roll-up scores” [56].

Additionally, studies have found that over 90% of websites do not include accessibility features for individuals with disabilities, such as hearing, cognitive, neurological, motor, language, or visual impairments [51,57,58]. Therefore, considering accessibility benefits not only for disabled users but also the general population (eg, those using mobile devices, with slow internet, or limited bandwidth), it provides equal access to information for elderly individuals or residents in remote areas [51,57,58].

#### Dashboard

In our sample, a lot of discussion revolved around dashboards. We see them as a subcategory of web-based information, offering more dynamic and versatile analysis possibilities than static websites.

Overall, 20% (5/25) of studies examined the presentation of information on dashboards. Relevant and high-quality data are crucial for constructing dashboards, and research indicates that data sources are key factors in dashboard development [59,60]. Dashboards are especially useful in presenting epidemic and pandemic trends, such as those of COVID-19 [61], but can also be used in classical resource selection performed by PR users.

In terms of content presentation, dashboards should serve as tools that clearly link current trends with past policy decisions and individual behaviors, thereby enhancing the use of indicators [61]. However, evidence suggests that known and validated presentation techniques were overall underused, especially explanatory narratives [61]. To enhance usability, effective dashboards include: (1) understanding the audience and their information needs; (2) managing the type, quantity, and flow of displayed information; (3) clearly reporting data sources and methods; (4) linking time trends with policy decisions; (5) providing “close-to-home” data; (6) segmenting populations into relevant subgroups; and (7) using storytelling and visual cues [61].

Among these studies, the predominant approach involved data provision (specificity of geographic breakdowns, range of indicators reported, and explanations of data sources or calculations) and advancements enabled by the technologies employed (customization of time trends and interactive or visual chart elements) [53]. Meanwhile, studies also note that the clarity of data sources and methods has improved in dashboard development [53]. Teams differ in how they explore and define the purposes of dashboards. Some view dashboards as tools for presenting raw data to the public, while others attempt to provide explanations through narrative or visualization [59].

Moreover, 8% (2/25) of studies viewed the purpose of dashboards as solely presenting data (raw numbers) for the public to interpret independently, whereas others sought to provide explanations through narrative or visual methods [53]. Additionally, incorporating storytelling elements and visual cues within dashboards has been shown to enhance users’ ability to process information effectively [53]. For example, the development of dashboards that allow users to create profiles and receive personalized scores enables consumers to “see themselves in the data” [62].

### Process

#### Data Quality

Overall, 48% (12/25) of studies examined PR from the perspective of website data. Notably, 32% (8/25) of studies indicated that standardized data guidelines help improve the quality of PR websites [15,45-47,58,59]. Additionally, some research described implementing check types within a data quality framework covering conformance, completeness, and plausibility, including both verification and validation [63].

#### Data Process

In the context of PPC, the generation of data encompasses multiple sources and activities. By using the data quality framework, data were collected across 4 domains, multiple measures, and time periods to examine access and equity; efficiency and sustainability; quality, safety, and patient orientation; and employee engagement [60].

Among these studies, 28% (7/25) investigated chart information on PR websites. The use of publicly reported data by consumers, policymakers, and researchers has grown substantially [64]. Most websites use charts, graphical elements, and quantitative descriptions as tools to convey differences between hospitals [65]. In addition, some research suggests adding charts to improve navigation [15]. For example, appropriate disease-related images and illustrations help patients better understand the information [50].

Meanwhile, the trend toward simplified composite rating systems may be reasonable to improve consumer responses to PR [55]. Previous research identified seven key elements that enhance user understanding [7]. The elements include unmarked bar charts, bar charts with simple symbols, bar charts with traffic light indicators, bar charts with thumbs-up symbols, provider rankings based on performance, clearly indicating whether higher values represent better performance, and implying quality levels through performance ranges without explicitly marking scale directions [7].

Furthermore, the transparent communication of standardized databases provides a crucial supplement to the missing components commonly observed in observational research [63]. This transparency can enhance the effectiveness of PR tools, as consumers may question the reliability of the quality information provided and consequently avoid using report cards [66].

Within the PPC process, presenting data in a structured format on dashboards can facilitate clear communication between health care providers and patients [67]. Meanwhile, PR websites can also serve as supplementary materials recommended by physicians, thereby reducing the need for face-to-face education and consultation [50].

### Moderating Variable

#### User Heterogeneity

Overall, 20% (5/25) of the studies examined user heterogeneity, suggesting that themes such as simplicity, trust, collaboration, software and data integration, and adaptability have facilitated the development of PR systems [67]. However, the use of identical data across different websites may result in confusion or even contradictions regarding the same provider [15].

Moreover, research has highlighted that improvements in data timeliness, the level of detail available, and the categorization of comparable population groups can enhance the quality and effectiveness of PR [60]. For instance, individuals with higher health literacy tend to spend more time reviewing PR information than those with lower health literacy [68].

#### Individual Characteristics and Contextual Factors

Similarly, individual characteristics and contextual factors, such as age, political orientation, employment status, income level, and residential area, significantly influence users’ adoption of PR information [38]. Younger adults are generally more likely than older adults to trust information from all sources [38]. In addition, political orientation, employment status, income level, and residential location are correlated with trust in medical quality information from companies, government agencies, or family and social network sources [38]. Collectively, these findings indicate that both individual characteristics and contextual factors are critical determinants of the perception of PR information.

### Outcome

#### Patient Satisfaction

Overall, although patients generally prefer to obtain health information from medical professionals, the media remains an important source of information [69]. Within the PPC process, patient satisfaction primarily depends on the quality of care and service delivery. Specifically, patient satisfaction appears to depend largely on postoperative pain levels and access to timely appointments [70].

#### Surgical Mortality

In addition, studies have pointed out that the variables influencing patient risk levels in surgical mortality include demographic factors such as age, weight, and prematurity, surgical factors such as the type of procedure, as well as disease-related factors [71].

### A Framework to Understand the PR Quality Indicators in Health Care

**Figure 2.**
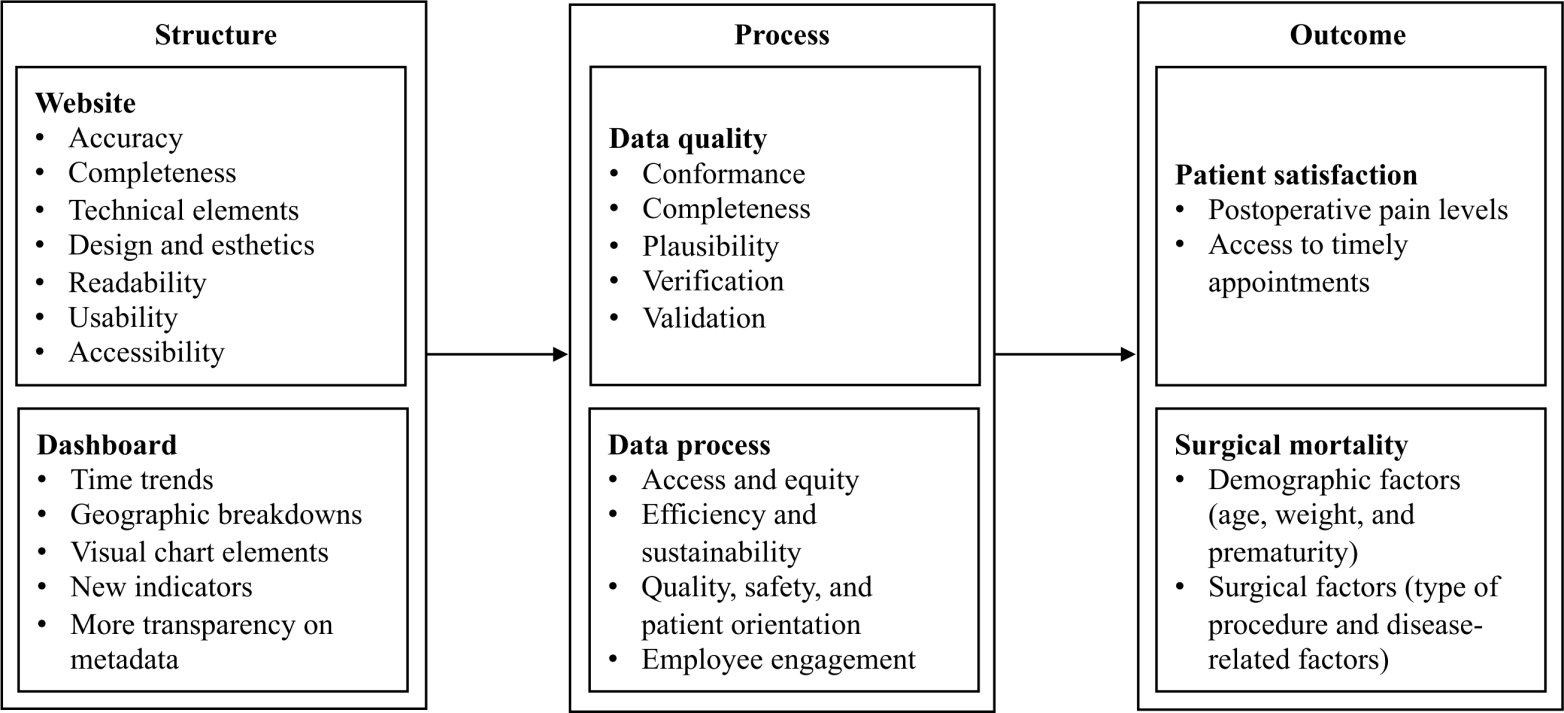
Integrative structure-process-outcome (SPO) framework for public reporting systems in health care.

The framework used in this study is based on the Donabedian quality model [20]. As shown in Figure 2, the system constitutes a key component of structural indicators [20]. User heterogeneity functions as a mediating mechanism linking the structural indicators and process indicators to outcome indicators. Specifically, the structural indicators and process indicators features of PR systems jointly shape users’ perception of the information [38].

The presentation format of PR websites refers to the channel through which users directly interact with the displayed content of the PR systems. Adherence to the website quality dimension and the dashboard quality dimension in the presentation format facilitates users’ understanding of the system in a structured context.

Previous studies have indicated that data inconsistencies negatively affect the actual use of the system, thereby influencing users’ adoption of the system [72]. The reviewed study indicates that the dashboards present data that have been processed and integrated within the underlying data architecture, resulting in higher accuracy and consistency. This, in turn, helps users use information that is more reliable and precise.

While structural indicators define the foundational quality of PR websites, process indicators determine how these systems function and generate reliable data. In terms of process indicators, data quality encompasses conformance, completeness, plausibility, verification, and validity. Data processes include accessibility and equity, efficiency and sustainability, quality, safety, patient-centered orientation, as well as employee engagement.

Building on the research gap regarding data inconsistency in existing PR, this study examines the dimensional indicators of data generated within process metrics. Standardizing data generated through PPC processes facilitates process-level data handling, thereby supporting the further use of the data. This aligns with prior findings from reviewed studies indicating that standardized data guidelines help users make better-informed health decisions [51].

User heterogeneity consists of individual characteristics and contextual design features [37]. Prior research suggests that dashboard design often fails to account for distinct user groups, leading to limited engagement [59]. Integrating contextual factors, health literacy levels, and individual characteristics into the design of PR systems allows for a more targeted presentation of information, thereby enhancing user comprehension and decision-making [37,59,68].

Previous research has shown that demographic variations influence information perception [73]. In line with this evidence, the reviewed studies identify multiple factors associated with users’ interactions with PR systems, including age, gender, political orientation, employment status, and region of residence. Beyond demographic variations, health literacy and contextual design features jointly shape how users interpret and use PR data [37].

The reviewed studies have demonstrated that audiovisual and narrative information can positively influence satisfaction measures; contextual features such as storytelling techniques, interactivity, and visual chart elements enhance user understanding [37]. Health literacy plays a critical role in users’ ability to interpret PR data. The reviewed studies found that individuals with higher health literacy are better able to comprehend PR information, which may explain their longer visual attention to such content [68]. This suggests that health literacy influences both the depth and quality of patient engagement in PPC processes. To address this, design features such as explanatory narratives [59], temporal trends and interactive functions [57], and appropriate visual chart elements can assist users with varying levels of health literacy in better understanding PR information [68].

This study further notes that within PR, patient satisfaction is primarily affected by postoperative pain levels (higher pain correlates with lower satisfaction) and access to timely appointments [70], consistent with previous findings linking health status to user satisfaction. Meanwhile, demographic factors (age, weight, and prematurity) and surgical factors (type of procedure and disease-related factors) influence surgical mortality [71].

By mapping the reviewed studies to the SPO framework, we illustrate that system data are no longer confined solely to structural indicators; they also play a role in process and outcome indicators. Accordingly, this study identifies website guidelines and dashboard guidelines under structural indicators, data quality and data processes under process indicators, and patient satisfaction and surgical mortality under outcome indicators. Furthermore, the underlying database sources are displayed within dashboards, helping to address inconsistencies between databases across different systems.

## Discussion

### Principal Findings

Health technology assessment ensures that medical innovations genuinely improve patient outcomes rather than merely increasing the burden [74]. In this literature review, we synthesized the findings of 25 empirical studies by categorizing them under structural indicators, process indicators, and outcome indicators [20]. Our main findings indicate that structural indicators (website and dashboard guidelines) and process indicators (data quality and data processes) jointly influence user outcomes, such as satisfaction and surgical mortality, with user heterogeneity mediating these effects.

This study adopts a dual perspective of the individual and the system in selection pathways to systematically examine the mechanisms through which information presentation and user interaction operate within PR systems [19]. Existing literature indicates that the presentation of information within information systems not only supports patients in selecting health care providers and using report cards [68] but also influences the quality of user decision-making [55]. Consistent with prior research, the mode of information presentation, the level of aggregation, and report personalization significantly affect the extent of user engagement in decision-making [7,62]. However, this study finds that the operability and data-display characteristics of dashboards play a unique role in supporting individualized decisions [53,61] and that the supplementation of online information can enhance users’ understanding of the data [51]. Additionally, the personal and contextual characteristics of health care information sources affect users’ adoption in PR systems [37].

Regarding consistency, most studies emphasize the importance of PR dashboards for user decision-making [67], while user health literacy and individual characteristics modulate actual information use [68]. This study further highlights that the standardized design of data and websites can enhance decision-making effectiveness [60]. Unified dashboard guidelines, along with aggregation and search functionalities, can reduce information conflicts and increase user comprehension and trust [59,68].

In terms of heterogeneity, studies have examined the impact of different information types (eg, online reviews, report cards, and dashboards during the pandemic) and diverse user groups (classified by age and health status) on decision-making [54]. Specifically, users differ in their focus on data operability versus information sources [37,61]. Common challenges in PR include missing information, delayed updates, complex formats, lack of standardization, and limited accessibility for users with low health literacy [51,68].

Regarding the user selection pathway, the system is considered a critical avenue for decision-making. Dashboards can mitigate existing issues in PR, such as missing data, delayed updates, and information confusion. As Donabedian [20] noted, while quality indicators are difficult to measure, their establishment contributes to improving actual health care quality. Standardized data guidelines and structured information presentation can support more effective decisions within structural and process indicators, while reducing information asymmetry and enhancing the quality of health services [55].

In summary, by referencing different PR scenarios and information types, this study proposes practical system optimization recommendations and categorizes differences among user groups as user-consciousness heterogeneity, which serves as a moderating variable. It emphasizes the role of standardized guidelines in facilitating the flow of information.

### Future Research Agenda

#### Overview

In the era of digital health, the adoption of PR has evolved. From the perspective of availability, it is possible to examine the innovativeness of technologies in shaping users’ perceptions of what they can accomplish [75]. Information systems, as a critical component of structural indicators [20], also play a significant role in enhancing patient awareness [15]. Based on the classification of diverse user groups and the multiple roles involved in the PPC process, incorporating dashboards and other visualization tools into system design can facilitate the adoption of PR information.

Based on the analysis of the studies included in this review, the public’s understanding and use of PR remain at a preliminary stage across different population groups [76,77]. To advance the practical use of PR information, future studies should adopt diverse methodological and theoretical approaches to explore how enhancing awareness can foster trust between health care organizations and various demographic groups. Research on this topic should be further expanded along four key dimensions: conceptual understanding, thematic exploration, methodological innovation, and technological development.

#### Conceptual Agenda

User attitudes are fundamental determinants of behavioral intentions and actual behaviors [17]. Underlying these attitudes are user cognitions, particularly awareness and understanding, which align with levels 1 and 2 of the PAM. While existing research highlights the role of health literacy in PR and its connection to PAM, little attention has been paid to its functional and interactive dimensions [78].

Although system design and information presentation can foster awareness and literacy, the conceptual link between the two remains underexplored. From a systems perspective, perception marks the initial awareness stage; subsequent information processing drives decision-making and system adoption. Information framing, as shown in media studies, shapes comprehension and interpretation [79]. Empirical evidence shows that most patients remain at level 1 of PAM [10]. Enhancing health literacy may help users progress from basic awareness to initial engagement.

#### Thematic Agenda

This study notes that participants with more severe health conditions tend to seek care more frequently than healthier individuals, making the process of choosing a physician particularly important for them. Notably, significant differences in decision quality are observed among these participants [56]. Previous research on the design of PR websites has largely focused on conditions such as hypertension, diabetes, and infectious diseases [15]. The future PR system should pay greater attention to the needs of patients with complex conditions, such as those with multiple comorbidities.

Moreover, this study points out that contextual factors, health literacy, and individual characteristics affect an individual’s adoption. Future research should explore how individuals’ emotional states and literacy capacities interact with and interpret PR information. For instance, Shaller [80] proposed a context-based consumer choice framework that emphasizes the integration of users’ decision contexts, emotional states, and literacy capacities.

#### Methodological Agenda

Primary data collection was primarily used in surveys, experiments, and semistructured interviews, whereas qualitative approaches, such as interviews, focus groups, and thematic content analysis, remained less common. Mixed method studies were rare, typically combining expert evaluation with descriptive and thematic analyses.

Methodologically combining natural language processing, causal inference, and experimental designs could advance the understanding of how system-level indicators interact with user heterogeneity, health literacy, and decision-making behaviors in the PR system.

#### Technological Agenda

In health care organizations, the PPC extends beyond physicians and patients to include family members, friends, caregivers, and other stakeholders. Future system designs that integrate and accommodate these multiple roles could enhance communication between actual users and the system.

Previous studies have indicated that patients with poorer health conditions exhibit more pronounced differences in decision-making [56]. Population segmentation can be categorized into low-health-demand, multiple comorbidities, medically complex, and frail groups [77]. Future research could explore the heterogeneity of responses across these groups in relation to information presentation. For example, web design could incorporate switchable versions to accommodate the health information needs of patients with different health conditions.

### Limitations

First, this study retrieved literature through keyword searches within selected databases. This approach may have limited the scope of study selection and potentially excluded relevant studies from other databases.

Second, this study explored PR systems from the perspective of individuals in health care. Even though employees act as individual users of the PR system, broadening the search term to include employees of health care organizations in their dual roles can bring new insights. It can be helpful to explore the interaction between employees in the organization and individuals within the sociotechnical theory. Additionally, given that qualitative research methods are interpretive, potential subjective biases may exist when evaluating study findings.

### Conclusions

This study provides the following contributions to the existing literature. First, we synthesized within the SPO framework. By systematically analyzing current research on the use of PR information presentation, this study provides an overview of the website and dashboard dimensions in the structure indicators, data quality and data process in the process indicators, and patient satisfaction and surgical mortality in the outcome indicators. Moreover, this study contributes to the literature by identifying contextual factors, such as health literacy, individual characteristics, political affiliation, employment status, income, and area of residence that influence users’ perceptions.

Second, this study builds a connection between data appearing during PPC and the PR systems in the structure quality dimension. Finally, this research outlines future research directions from conceptual, methodological, thematic, and information system perspectives. These insights provide valuable guidance for advancing research in this field, bridging existing gaps, and improving the overall quality of academic inquiry.

## Supplementary material

10.2196/80435 Multimedia Appendix 1 Search strategies in each database.

10.2196/80435 Multimedia Appendix 2 Mixed Methods Appraisal Tool (MMAT) quality assessment report.

10.2196/80435 Multimedia Appendix 3 Key characteristics of the included studies.

10.2196/80435 Multimedia Appendix 4 Details of the included studies.

10.2196/80435 Checklist 1 PRISMA-P checklist.

## Abbreviations

EC: exclusion criteria
ESG: environmental, social, and governance
IC: inclusion criteria
MMAT: Mixed Methods Appraisal Tool
PAM: patient activation measure
PPC: patient-physician collaboration
PR: public reporting
PRISMA: Preferred Reporting Items for Systematic Reviews and Meta-Analyses
PRISMA-P: Preferred Reporting Items for Systematic Review and Meta-Analyses Protocols
SPO: structure-process-outcome
