# Supplementary material for: Digital Public Reporting Systems for Evaluating Health Care Quality: Systematic Review
Source: JMIR Med Inform. 2026 Mar 18;14:e80435. doi: 10.2196/80435 (PMC12998539; doi:10.2196/80435)
Supplement: Multimedia Appendix 1 [file medinform-v14-e80435-s001.docx]

**Appendix 1.** **Search strategies in each database**

("information" OR "data display" OR "information display" OR "information visualization" OR "data visualization" OR "data interpretation")

AND

(patient* OR consumer* OR user* OR public*)

AND

(website* OR dashboard* OR interface* OR visual* OR "web page*" OR "online report*")

AND

("public reporting" OR "performance reporting" OR "quality reporting" OR "hospital report card*" OR "comparative health report*")

AND

("health care" OR healthcare OR hospital* OR clinic* OR medical)

Search time: September 2025

Search strategies in five databases:

--------------------------------------------------------------------------------

| Database | Search terms |
| --- | --- |
| PsycInfo (10) | DE "Data Visualization" OR DE "Information Display" OR DE "Data Display" OR DE "Information Interpretation"  OR TI ("information" OR "data display" OR "information display" OR "information visualization" OR "data visualization" OR "data interpretation")  OR AB ("information" OR "data display" OR "information display" OR "information visualization" OR "data visualization" OR "data interpretation")  AND  DE "Patient" OR DE "Consumer" OR DE "User" OR DE "Public"  OR TI (patient* OR consumer* OR user* OR public*)  OR AB (patient* OR consumer* OR user* OR public*)  AND  TI (website* OR dashboard* OR interface* OR visual* OR "web page*" OR "online report*")  OR AB (website* OR dashboard* OR interface* OR visual* OR "web page*" OR "online report*")  AND  DE "Public Reporting" OR DE "Performance Reporting" OR DE "Quality Reporting" OR DE "Hospital Report Card" OR DE "Comparative Health Report"  OR TI ("public reporting" OR "performance reporting" OR "quality reporting" OR "hospital report cards" OR "comparative health reports")  OR AB ("public reporting" OR "performance reporting" OR "quality reporting" OR "hospital report cards" OR "comparative health reports")  AND  DE "Health Care" OR DE "Hospital" OR DE "Clinic" OR DE "Medical"  OR TI (healthcare OR "health care" OR hospital* OR clinic* OR medical)  OR AB (healthcare OR "health care" OR hospital* OR clinic* OR medical) |
| EMBASE (126) | 'data visualization'/exp OR 'information display'/exp OR 'data display'/exp OR 'information interpretation'/exp OR ("information":ab,ti OR "data display":ab,ti OR "information display":ab,ti OR "information visualization":ab,ti OR "data visualization":ab,ti OR "data interpretation":ab,ti) AND 'patient'/exp OR 'consumer'/exp OR 'user'/exp OR 'public'/exp OR (patient* OR consumer* OR user* OR public*):ab,ti AND (website* OR dashboard* OR interface* OR visual* OR "web page*" OR "online report*"):ab,ti AND 'public reporting'/exp OR 'performance reporting'/exp OR 'quality reporting'/exp OR 'hospital report card'/exp OR 'comparative health report'/exp OR ("public reporting" OR "performance reporting" OR "quality reporting" OR "hospital report cards" OR "comparative health reports"):ab,ti AND ('health care'/exp OR 'hospital'/exp OR 'clinic'/exp OR 'medical'/exp OR healthcare:ab,ti OR "health care":ab,ti OR hospital*:ab,ti OR clinic*:ab,ti OR medical:ab,ti) |
| PubMed (3) | (("data visualization"[MeSH Terms] OR "information display"[tiab] OR "data display"[tiab] OR "information visualization"[tiab] OR "data visualization"[tiab] OR "data interpretation"[tiab] OR "information interpretation"[tiab])  AND  ("patient"[MeSH Terms] OR "consumer"[tiab] OR "user"[tiab] OR "public"[tiab] OR patient*[tiab] OR consumer*[tiab] OR user*[tiab] OR public*[tiab])  AND  (website*[tiab] OR dashboard*[tiab] OR interface*[tiab] OR visual*[tiab] OR "web page*"[tiab] OR "online report*"[tiab])  AND  ("public reporting"[tiab] OR "performance reporting"[tiab] OR "quality reporting"[tiab] OR "hospital report cards"[tiab] OR "comparative health reports"[tiab])  AND  ("Health Care"[MeSH Terms] OR "Hospitals"[MeSH Terms] OR "Clinics, Hospital"[MeSH Terms] OR healthcare[tiab] OR "health care"[tiab] OR hospital*[tiab] OR clinic*[tiab] OR medical[tiab])) |
| Web of science (44) | (TI=("information" OR "data display" OR "information display" OR "information visualization" OR "data visualization" OR "data interpretation")  OR AB=("information" OR "data display" OR "information display" OR "information visualization" OR "data visualization" OR "data interpretation"))  AND  (TI=(patient* OR consumer* OR user* OR public*)  OR AB=(patient* OR consumer* OR user* OR public*))  AND  (TI=(website* OR dashboard* OR interface* OR visual* OR "web page*" OR "online report*")  OR AB=(website* OR dashboard* OR interface* OR visual* OR "web page*" OR "online report*"))  AND  (TI=("public reporting" OR "performance reporting" OR "quality reporting" OR "hospital report card*" OR "comparative health report*")  OR AB=("public reporting" OR "performance reporting" OR "quality reporting" OR "hospital report card*" OR "comparative health report*"))  AND (TI=(healthcare OR "health care" OR hospital* OR clinic* OR medical)  OR AB=(healthcare OR "health care" OR hospital* OR clinic* OR medical)) |
| CINAHL (29) | ((MH "Data Visualization+") OR (MH "Information Display+") OR (MH "Data Display+") OR (MH "Information Interpretation+") OR (TI "information" OR TI "data display" OR TI "information display" OR TI "information visualization" OR TI "data visualization" OR TI "data interpretation" OR AB "information" OR AB "data display" OR AB "information display" OR AB "information visualization" OR AB "data visualization" OR AB "data interpretation"))  AND  ((MH "Patients") OR (MH "Consumers") OR (MH "Users") OR (MH "Public") OR (TI patient* OR TI consumer* OR TI user* OR TI public* OR AB patient* OR AB consumer* OR AB user* OR AB public*))  AND  (TI website* OR TI dashboard* OR TI interface* OR TI visual* OR TI "web page*" OR TI "online report*" OR AB website* OR AB dashboard* OR AB interface* OR AB visual* OR AB "web page*" OR AB "online report*")  AND  ((MH "Public Reporting") OR (MH "Performance Reporting") OR (MH "Quality Reporting") OR (MH "Hospital Report Cards") OR (MH "Comparative Health Reports") OR (TI "public reporting" OR TI "performance reporting" OR TI "quality reporting" OR TI "hospital report cards" OR TI "comparative health reports" OR AB "public reporting" OR AB "performance reporting" OR AB "quality reporting" OR AB "hospital report cards" OR AB "comparative health reports"))  AND  ((MH "Health Care") OR (TI healthcare OR TI "health care" OR TI hospital* OR TI clinic* OR TI medical OR AB healthcare OR AB "health care" OR AB hospital* OR AB clinic* OR AB medical)) |
