# Supplementary material for: Digital Public Reporting Systems for Evaluating Health Care Quality: Systematic Review
Source: JMIR Med Inform. 2026 Mar 18;14:e80435. doi: 10.2196/80435 (PMC12998539; doi:10.2196/80435)
Supplement: Multimedia Appendix 2 [file medinform-v14-e80435-s002.docx]

**Appendix 2. Mixed Methods Appraisal Tool (MMAT) quality assessment report**

**Table 1. Mixed Methods Appraisal Tool (MMAT), version 2018.**

| **Category of study designs** | **Methodological quality criteria** |
| --- | --- |
|  |  |
| Screening questions  (for all types) | S1. Are there clear research questions? |
|  | S2. Do the collected data allow to address the research questions? |
| 1. Qualitative method | 1.1. Is the qualitative approach appropriate to answer the research question? |
|  | 1.2. Are the qualitative data collection methods adequate to address the research question? |
|  | 1.3. Are the findings adequately derived from the data? |
|  | 1.4. Is the interpretation of results sufficiently substantiated by data? |
|  | 1.5. Is there coherence between qualitative data sources, collection, analysis and interpretation? |
| 2. Quantitative randomized controlled trials | 2.1. Is randomization appropriately performed? |
|  | 2.2. Are the groups comparable at baseline? |
|  | 2.3. Are there complete outcome data? |
|  | 2.4. Are outcome assessors blinded to the intervention provided? |
|  | 2.5 Did the participants adhere to the assigned intervention? |
| 3. Quantitative non-randomized trials | 3.1. Are the participants representative of the target population? |
|  | 3.2. Are measurements appropriate regarding both the outcome and intervention (or exposure)? |
|  | 3.3. Are there complete outcome data? |
|  | 3.4. Are the confounders accounted for in the design and analysis? |
|  | 3.5. During the study period, is the intervention administered (or exposure occurred) as intended? |
| 4. Quantitative descriptive trials | 4.1. Is the sampling strategy relevant to address the research question? |
|  | 4.2. Is the sample representative of the target population? |
|  | 4.3. Are the measurements appropriate? |
|  | 4.4. Is the risk of nonresponse bias low? |
|  | 4.5. Is the statistical analysis appropriate to answer the research question? |
| 5. Mixed methods | 5.1. Is there an adequate rationale for using the mixed methods design to address the research question? |
|  | 5.2. Are the different components of the study effectively integrated to answer the research question? |
|  | 5.3. Are the outputs of the integration of qualitative and quantitative components adequately interpreted? |
|  | 5.4. Are divergences and inconsistencies between quantitative and qualitative results adequately addressed? |
|  | 5.5. Do the different components of the study adhere to the quality criteria of each tradition of the methods involved? |
| Note: To appraise 5.5, use criteria for the qualitative component (1.1-1.5), and the appropriate criteria for the quantitative component (2.1-2.5, or 3.1-3.5, or 4.1-4.5). | |

**Table 2. Quality of the included studies (n=25)**

|  | **Reference** | **Qualitative** | | | | | **Quantitative RCTs** | | | | | **Quantitative nonrandomized** | | | | | **Quantitative descriptive** | | | | | **Mixed methods** | | | | | **Total score** |
| --- | --- | --- | --- | --- | --- | --- | --- | --- | --- | --- | --- | --- | --- | --- | --- | --- | --- | --- | --- | --- | --- | --- | --- | --- | --- | --- | --- |
|  |  | 1 | 2 | 3 | 4 | 5 | 1 | 2 | 3 | 4 | 5 | 1 | 2 | 3 | 4 | 5 | 1 | 2 | 3 | 4 | 5 | 1 | 2 | 3 | 4 | 5 |  |
|  | (1) | Y | - | Y | - | Y | - | - | - | - | - | - | - | - | - | - | - | - | - | - | - | - | - | - | - | - | 3 |
|  | (2) | - | - | - | - | - | - | - | - | - | - | - | Y | Y | - | Y | - | - | - | - | - | - | - | - | - | - | 3 |
|  | (3) | - | - | - | - | - | - | - | - | - | - | - | - | - | - | - | Y | - | Y | - | Y | - | - | - | - | - | 3 |
|  | (4) | - | - | - | - | - | - | - | - | - | - | - | - | - | - | - | Y | - | Y | - | Y | - | - | - | - | - | 3 |
|  | (5) | Y | Y | Y | Y | Y | - | - | - | - | - | - | - | - | - | - | - | - | - | - | - | - | - | - | - | - | 5 |
| delete | (6) | - | - | - | - | - | Y | - | - | - | - | - | - | - | - | - | - | - | - | - | - | - | - | - | - | - | 1 |
|  | (7) | - | - | - | - | - | - | - | - | - | - | - | - | - | - | - | Y | - | Y | - | Y | - | - | - | - | - | 3 |
|  | (8) | - | - | - | - | - | - | - | - | - | - | - | - | - | - | - | Y | - | Y | Y | Y | - | - | - | - | - | 4 |
|  | (9) | - | - | - | - | - | - | - | - | - | - | - | - | - | - | - | Y | - | Y | Y | Y | - | - | - | - | - | 4 |
|  | (10) | - | - | - | - | - | - | - | - | - | - | - | - | - | - | - |  | - |  |  |  | Y | Y | Y | - | Y | 4 |
|  | (11) | - | - | - | - | - | - | - | - | - | - | - | - | - | - | - | - | - | Y | Y | Y | - | - | - | - | - | 3 |
|  | (12) | - | - | - | - | - | - | - | - | - | - | - | - | - | - | - | Y | - | Y | Y | Y | - | - | - | - | - | 4 |
|  | (13) | - | - | - | - | - | - | - | - | - | - | - | - | - | - | - |  | - |  |  |  | Y | Y | Y | Y | Y | 5 |
|  | (14) | Y | Y | Y | Y | Y | - | - | - | - | - | - | - | - | - | - | - | - | - | - | - | - | - | - | - | - | 5 |
|  | (15) | - | - | - | - | - | - | - | - | - | - | Y | - | Y | - | Y | - | - | - | - | - | - | - | - | - | - | 3 |
|  | (16) | - | - | - | - | - | - | - | - | - | - | Y | Y | Y | - | Y | - | - | - | - | - | - | - | - | - | - | 4 |
|  | (17) | - | - | - | - | - | - | - | - | - | - | Y | Y | Y | - | Y | - | - | - | - | - | - | - | - | - | - | 4 |
|  | (18) | - | - | - | - | - | - | - | - | - | - | Y | Y | Y | - | Y | - | - | - | - | - | - | - | - | - | - | 4 |
|  | (19) | - | - | - | - | - | Y | Y | Y | - | Y | - | - | - | - | - | - | - | - | - | - | - | - | - | - | - | 4 |
|  | (20) | - | - | - | - | - | - | - | - | - | - | - | - | - | - | - | Y | - | Y | - | Y | - | - | - | - | - | 3 |
|  | (21) | - | - | - | - | - | - | - | - | - | - | - | - | - | - | - | Y | - | Y | Y | Y | - | - | - | - | - | 4 |
| delete | (22) | - | - | - | - | - | - | - | - | - | - | - | - | - | - | - | - | Y | Y | - | - | - | - | - | - | - | 2 |
|  | (23) | - | - | - | - | - | - | - | - | - | - | - | - | - | - | - | Y | Y | Y | Y | Y | - | - | - | - | - | 5 |
|  | (24) | - | - | - | - | - | - | - | - | - | - | - | - | - | - | - | - | Y | Y | - | Y | - | - | - | - | - | 4 |
|  | (25) | - | - | - | - | - | - | - | - | - | - | - | - | - | - | - | Y | Y | Y | - | Y | - | - | - | - | - | 4 |
|  | (26) | Y | - | Y | Y | - | - | - | - | - | - | - | - | - | - | - | - | - | - | - | - | - | - | - | - | - | 3 |
|  | (27) | - | - | - | - | - | - | - | - | - | - | - | - | - | - | - | Y | Y | Y | - | Y | - | - | - | - | - | 4 |

1. Sapin M, Ehlig D, Geissler A, Vogel J. Public reporting in five health care areas: A comparative content analysis across nine countries. Health Policy. 2025;152:105222.

2. Yilmaz NG, Timmermans DR, Van Weert JC, Damman OC. Breast cancer patients' visual attention to information in hospital report cards: An eye-tracking study on differences between younger and older female patients. HEALTH INFORMATICS JOURNAL. 2023;29(1).

3. Temkin-Greener H, Mao Y, McGarry B. Online Customer Reviews of Assisted Living Communities: Association with Community, County, and State Factors. Journal of the American Medical Directors Association. 2023;24(6):841-5. e3.

4. Kast K, Otten S-M, Konopik J, Maier CB. Web-Based Public Reporting as a Decision-Making Tool for Consumers of Long-Term Care in the United States and the United Kingdom: Systematic Analysis of Report Cards. JMIR Formative Research. 2023;7:e44382.

5. Gephart SM, Tolentino DA, Quinn MC, Wyles C. Neonatal intensive care workflow analysis informing NEC-zero clinical decision support design. CIN: Computers, Informatics, Nursing. 2023;41(2):94-101.

6. Yilmaz NG, Timmermans DRM, Portielje J, Van Weert JCM, Damman OC. Testing the effects on information use by older versus younger women of modality and narration style in a hospital report card. Health Expectations. 2022;25(2):567-78.

7. Shah NS, Umeda Y, Newyear B, Matar RN, Frederickson M, Parman MD, et al. Patients with higher postoperative pain after ambulatory shoulder surgery reported lower satisfaction: a prospective observational study. Ame Surgical Journal. 2022;2.

8. Lopez-Olivo MA, des Bordes JK, Syed MN, Alemam A, Dodeja A, Abdel-Wahab N, et al. Quality appraisal of educational websites about osteoporosis and bone health. Archives of Osteoporosis. 2021;16(1):28.

9. Lloyd S, Cliff C, FitzGerald G, Collie J. Can publicly reported data be used to understand performance in an Australian rural hospital? Health Information Management Journal. 2021;50(1-2):35-46.

10. Ivanković D, Barbazza E, Bos V, Brito Fernandes Ó, Jamieson Gilmore K, Jansen T, et al. Features constituting actionable COVID-19 dashboards: descriptive assessment and expert appraisal of 158 public web-based COVID-19 dashboards. Journal of medical Internet research. 2021;23(2):e25682.

11. Hennessee I, Clennon JA, Waller LA, Kitron U, Bryan JM. Considerations for improving reporting and analysis of date-based COVID-19 surveillance data by public health agencies. American Journal of Public Health. 2021;111(12):2127-32.

12. Blacketer C, Defalco FJ, Ryan PB, Rijnbeek PR. Increasing trust in real-world evidence through evaluation of observational data quality. Journal of the American Medical Informatics Association. 2021;28(10):2251-7.

13. Barbazza E, Ivanković D, Wang S, Gilmore KJ, Poldrugovac M, Willmington C, et al. Exploring changes to the actionability of COVID-19 dashboards over the course of 2020 in the Canadian context: descriptive assessment and expert appraisal study. Journal of Medical Internet Research. 2021;23(8):e30200.

14. Barbazza E, Ivanković D, Davtyan K, Poldrugovac M, Yelgezekova Z, Willmington C, et al. The experiences of 33 national COVID-19 dashboard teams during the first year of the pandemic in the World Health Organization European Region: A qualitative study. Digital Health. 2022;8:20552076221121154.

15. Ryskina KL, Andy AU, Manges KA, Foley KA, Werner RM, Merchant RM. Association of online consumer reviews of skilled nursing facilities with patient rehospitalization rates. JAMA network open. 2020;3(5):e204682-e.

16. Moore AR, Hudson C, Amey F, Chumbler N. Trusting sources of information on quality of physician care. INQUIRY: The Journal of Health Care Organization, Provision, and Financing. 2020;57:0046958020952912.

17. Perraillon MC, Konetzka RT, He D, Werner RM. Consumer response to composite ratings of nursing home quality. American Journal of Health Economics. 2019;5(2):165-90.

18. Hsu SH, Hung P, Wang S-Y. Factors Associated With Hospices' Nonparticipation in Medicare's Hospice Compare Public Reporting Program. Medical Care. 2019;57(1):28-35.

19. Cerully JL, Parker AM, Rybowski L, Schlesinger M, Shaller D, Grob R, et al. Improving patients’ choice of clinician by including roll-up measures in public Healthcare quality reports: an online experiment. Journal of General Internal Medicine. 2019;34(2):243-9.

20. Walsh-Childers K, Braddock J, Rabaza C, Schwitzer G. One step forward, one step back: changes in news coverage of medical interventions. Health communication. 2018;33(2):174-87.

21. Siddhanamatha HR, Heung E, de los Angeles Lopez-Olivo M, Abdel-Wahab N, Ojeda-Prias A, Willcockson I, et al., editors. Quality assessment of websites providing educational content for patients with rheumatoid arthritis. Seminars in Arthritis and Rheumatism; 2017: Elsevier.

22. Pross C, Averdunk L-H, Stjepanovic J, Busse R, Geissler A. Health care public reporting utilization–user clusters, web trails, and usage barriers on Germany’s public reporting portal Weisse-Liste. de. BMC medical informatics and decision making. 2017;17:1-15.

23. Jacobs JP, editor The society of thoracic surgeons congenital heart surgery database public reporting initiative. Seminars in Thoracic and Cardiovascular Surgery: Pediatric Cardiac Surgery Annual; 2017: Elsevier.

24. Emmert M, Wiener M. What factors determine the intention to use hospital report cards? The perspectives of users and non-users. Patient education and counseling. 2017;100(7):1394-401.

25. Downing NS, Cloninger A, Venkatesh AK, Hsieh A, Drye EE, Coifman RR, et al. Describing the performance of US hospitals by applying big data analytics. PLoS One. 2017;12(6):e0179603.

26. Smith MA, Bednarz L, Nordby PA, Fink J, Greenlee RT, Bolt D, et al. Increasing consumer engagement by tailoring a public reporting website on the quality of diabetes care: a qualitative study. Journal of medical internet research. 2016;18(12):e332.

27. Reid RO, Deb P, Howell BL, Conway PH, Shrank WH. The roles of cost and quality information in Medicare Advantage plan enrollment decisions: an observational study. Journal of general internal medicine. 2016;31:234-41.
