# Supplementary material for: Digital Public Reporting Systems for Evaluating Health Care Quality: Systematic Review
Source: JMIR Med Inform. 2026 Mar 18;14:e80435. doi: 10.2196/80435 (PMC12998539; doi:10.2196/80435)
Supplement: Multimedia Appendix 3 [file medinform-v14-e80435-s003.docx]

**Appendix 3. Key characteristics of the included studies**

| **Reference** | **method** | **Data source** | **Method** | **Research question** | **Context** | **Main finding** | **Key Dimension** |
| --- | --- | --- | --- | --- | --- | --- | --- |
| [1] | qualitative | primary data | interviews | To understand how these websites support patients in making informed choices about healthcare providers. | Public reporting is crucial to enhance transparency, accountability, and informed provider choice. Therefore, providing accessible and reliable information on provider performance and activities is key for all healthcare areas and the utilization of information by patients, providers and related audiences. | The findings suggest that establishing national standards for public reporting may reduce the risk of presenting contradictory information to patients and thus, improve provider choice. | Process/Moderating variable |
| [2] | quantitative | primary data | empirical + survey | To (1) explore how women visually attend to a hospital report card (HRC), (2) explore whether visual attention of younger and older women (patients and non-patients) differs. | Visual attention to the hospital of choice (vs not of choice) and to indicators perceived as most important (vs least important) did not differ. However, women with higher health literacy looked longer at the HRC than women with lower health literacy. | Pre-existing conceptions of what information is relevant might result in more in-depth information processing among older patients than younger patients. In general, differences in level of health literacy, rather than (chronological) age, seem to be relevant to take into account when designing and/or updating HRCs. | Moderating variable |
| [3] | quantitative | secondary data | quantitative observational | online reviews as a possible source of information about these communities and examined the association between the reviews and aspects of state regulations, while controlling for assisted living, county, and state market-level factors. | Online reviews provided by users of assisted living communities may offer a unique source of heretofore unexamined data. | Online reviews are increasingly common, including in long-term care. These reviews are a promising source of information about important aspects of satisfaction, particularly in care settings that lack a public reporting infrastructure. | Structure |
| [4] | quantitative | secondary data | descriptive cross-sectional study | This study aims to examine the current state of web-based public reporting on long-term care facilities in the United States and the United Kingdom. | Report cards can help consumers make an informed decision when searching for a long-term care facility. | Apparently, the identified report cards have deficits. To make them more helpful for users and to bring public reporting a bit closer to its goal of improving the quality of health care services, both countries are advised to concentrate on optimizing the existing report cards. Those should become more transparent and improve the reporting of prices and consumer feedback. Advanced search, simplification tools, and comparison functions should be integrated more widely. | Process |
| [5] | qualitative | primary data | focus groups | The purpose of this study was to describe the current clinical workflow and sociotechnical processes among clinicians for necrotizing enterocolitis risk awareness, timely recognition of symptoms, and treatment to inform decision support design. | Decision support in neonatal ICUs is needed, especially for prevention and risk awareness of the devastating complication of necrotizing enterocolitis, a major cause of emergency surgery among fragile infants. | Clinicians desired (1) a necrotizing enterocolitis-relevant dashboard to support nutrition tracking and necrotizing enterocolitis recognition; (2) features to support decision-making (eg, necrotizing enterocolitis risk and adherence scoring); (3) breast milk tracking and feeding clinical decision support; (4) tools for necrotizing enterocolitis surveillance and quality reporting; and (5) general EHR optimizations to improve user experience. | Process |
| [6] | quantitative | primary data | survey | The purpose of this study was to evaluate satisfaction scores according to the CG-CAHPS and OAS-CAHPS surveys after outpatient shoulder surgery and their associated factors. | Due to the Affordable Care Act passed in 2010, the Consumer Assessment of Healthcare Providers and Systems (CAHPS) surveys have become a mandatory part of performance reporting. As the Center for Medicaid and Medicare Services (CMS) plans to extend their assessment of patient care from Clinician and Group (CG-CAHPS) to Outpatient Ambulatory Surgery (OAS-CAHPS), it becomes important to understand these two scores and their determinants. | Patients who had higher VAS scores for pain at time of discharge report lower levels of clinician (CG-CAHPS) and ambulatory surgery center (OAS-CAHPS) satisfaction after outpatient shoulder surgery. Overall, patient CAHPS satisfaction seems to be dependent on postoperative pain levels and access to timely appointments. | Outcome |
| [7] | quantitative | secondary data | descriptive cross-sectional study | Bone health education publicly available through the Internet, if evidence-based and unbiased, could help patients deal with issues such as decision-making, maintaining healthy lifestyles, using medications correctly, and improving their communication with health professionals. | Bone health education publicly available through the Internet, if evidence-based and unbiased, could help patients deal with issues such as decision-making, maintaining healthy lifestyles, using medications correctly, and improving their communication with health professionals. | Websites with information about bone health and osteoporosis commonly present information about initial. Almost half of all the websites we assessed did not provide sufficient information to determine the sources of evidence, or whether the recommendations were based on current guidelines or recent research. The main aspect of concern was completeness. Most websites lacked information about when the content was created and also failed to update their information on a yearly basis. This is vital to patients because they might be misled into reading about outdated information which may or may not be applicable to their current scenario. | Structure/Process |
| [8] | quantitative | secondary data | secondary data analysis and performance visualization | To determine whether publicly available information can be used to measure health service performance in a rural hospital | Despite agreement among policymakers, funders, consumers and researchers about the value of public reporting of health information, limited attention has been paid to how it can be used to understand the performance of rural hospitals | Publicly reported data can be used to represent performance for a rural hospital. Timeliness, level of detail available and peer groupings of data limits optimal utility. Consumers, clinicians and health service managers wanting to understand the performance of rural hospitals will need to use significant health information management skills to gain a picture of performance. | Process/Moderating variable |
| [9] | mixed method | secondary data | expert evaluation + descriptive analysis + Thematic analysis | The aim of this study is to explore the characteristics of public web-based COVID-19 dashboards by assessing their purpose and users (“why”), content and data (“what”), and analyses and displays (“how” they communicate COVID-19 data), and ultimately to appraise the common features of highly actionable dashboards. | Since the outbreak of COVID-19, the development of dashboards as dynamic, visual tools for communicating COVID-19 data has surged worldwide. Dashboards can inform decision-making and support behavior change. To do so, they must be actionable. The features that constitute an actionable dashboard in the context of the COVID-19 pandemic have not been rigorously assessed. | COVID-19 dashboards are diverse in the why, what, and how by which they communicate insights on the pandemic and support data-driven decision-making. To leverage their full potential, dashboard developers should consider adopting the seven actionability features identified. | Structure |
| [10] | quantitative | secondary data | observational / methodological analysis | The choice of which date to use should be guided by a balance between interpretability and epidemiological relevance. | More than a year after the first domestic COVID-19 cases, the United States does not have national standards for COVID-19 surveillance data analysis and public reporting. This has led to dramatic variations in surveillance practices among public health agencies, which analyze and present newly confirmed cases by a wide variety of dates. | Symptom onset date is better suited for such analyses because of its clinical and epidemiological relevance. However, using symptom onset for public reporting of new confirmed cases can cause confusion because reporting lags result in an artificial decline in recent cases. | Structure/Process |
| [11] | quantitative | secondary data | quantitative descriptive analysis | The Data Quality Dashboard is an open-source R package that reports potential quality issues in an OMOP CDM instance through the systematic execution and summarization of over 3,300 configurable data quality checks | Advances in standardization of observational healthcare data have enabled methodological breakthroughs, rapid global collaboration, and generation of real-world evidence to improve patient outcomes. Standardizations in data structure, such as use of Common Data Models (CDM), need to be coupled with standardized approaches for data quality assessment. | We discuss how data quality reporting can become part of the overall real-world evidence generation and dissemination process to promote transparency and build confidence in the resulting output. Transparently communicating how well CDM standardized databases adhere to a set of quality measures adds a crucial piece that is currently missing from observational research. Assessing and improving the quality of our data will inherently improve the quality of the evidence we generate. | Process |
| [12] | quantitative | secondary data | descriptive statistics + expert consensus discussion | We aimed to explore how COVID-19 dashboards evolved in the Canadian context during 2020 and whether the presence of actionability features changed over time. | Public web-based COVID-19 dashboards are in use worldwide to communicate pandemic-related information. Actionability of dashboards, as a predictor of their potential use for data-driven decision-making, was assessed in a global study during the early stages of the pandemic. | Improvements to COVID-19 dashboards in the Canadian context during 2020 were seen mostly in data availability and dashboard technology. Further improving the actionability of dashboards for public reporting will require attention to both technical and organizational aspects of dashboard development. | Structure |
| [13] | qualitative | primary data | semi-structured interviews | This study explores the development of COVID-19 dashboards during the first year of the pandemic and identifies common barriers, enablers and lessons from the experiences of teams responsible for their development. | Governments across the World Health Organization (WHO) European Region have prioritised dashboards for reporting COVID-19 data. The ubiquitous use of dashboards for public reporting is a novel phenomenon. | COVID-19 dashboards were developed in a learning-by-doing approach. The experiences of teams reveal that initial under preparedness was offset by high-level political endorsement, the professionalism of teams, accelerated data improvements and immediate support with commercial software solutions. To leverage the full potential of dashboards for health data reporting, investments are needed at the team, national and pan-European levels. | Moderating variable |
| [14] | quantitative | secondary data | linear regression + natural language processing | To assess the association between rehospitalization rates and online ratings of SNFs; to compare the association of rehospitalization with ratings from a review website vs Medicare Nursing Home Compare (NHC) rating; and to identify specific topics consistently reported in reviews of SNFs with the highest vs lowest rehospitalization rates using natural language processing. | There are areas of skilled nursing facility (SNF) experience of importance to the public that are not currently included in public reporting initiatives on SNF quality. Whether patients, hospitals, and payers can leverage the information available from unsolicited online reviews to reduce avoidable rehospitalizations from SNFs is unknown. | Skilled nursing facilities with the best rating on both a review website and NHC had slightly lower rehospitalization rates than SNFs with the best rating on NHC alone. However, there was marked variation in the volume of reviews, and many SNF characteristics were underrepresented. Further refinement of the review process is warranted. | Structure |
| [15] | quantitative | secondary data | factor analysis | Our aim is to understand the contextual and personal characteristics that influence patient-consumers’ decisions to trust or ignore information sources about healthcare quality. | Reporting healthcare quality has become an important factor in healthcare delivery. Prior research has shown that patient-consumers do not frequently use information on websites reporting physician quality to guide their choice of physicians. | The results indicate that individual and contextual characteristics are important factors in trusting information sources, independent of health status. These should be taken into consideration by advocates of public reporting. | Moderating variable |
| [16] | quantitative | secondary data | quantitative causal/analytic study | the form of the information may matter to consumers. | Health care report cards are intended to address information asymmetries and enable consumers to choose providers of better quality. | Our main results show that nursing homes that obtained an additional star gained more admissions, with heterogeneous effects depending on baseline number of stars. We conclude that the form of quality reporting matters to consumers, and that the increased use of composite ratings is likely to increase consumer response | Structure/Process |
| [17] | quantitative | secondary data | logistic regression | To examine the factors associated with hospices’ nonparticipation in Hospice Compare. | To enhance the quality of hospice care and to facilitate consumers’ choices, the Centers for Medicare and Medicaid Services (CMS) began the Hospice Quality Reporting Program, in which CMS posted the quality measures of participating hospices on its reporting website, *Hospice Compare*. Little is known about the participation rate and the types of nonparticipating hospices. | Hospice Compare successfully motivated hospice in participating in the quality report program in most of states. For-profit hospices, hospices with less quality, and hospices located in competitive markets were less likely to participate. Further research is warranted to examine the quality of these nonparticipants, especially in the 4 states with a lower participation rate. | Process |
| [18] | quantitative | primary data | experiment | To determine how presenting quality scores at different levels of aggregation affects patients’ clinician choices. | Public reports on healthcare quality typically include complex data. To lower the cognitive burden of interpreting these data, some report designers create summary, or roll-up, measures combining multiple indicators of quality into one score. Little is known about how the availability of roll-ups affects clinician choice. | Our results suggest that roll-ups in healthcare quality reports, alone or as a complement to drill-downs, can help patients make better decisions for themselves. | Structure |
| [19] | quantitative | secondary data | quantitative content analysis | The reviews offered a measure by which the public and journalists themselves could assess the completeness and usefulness of health coverage across 10 criteria for quality reporting. | During 2005–2013, the award-winning website HealthNewsReview.org offered reviews of major media outlets’ news stories related to health interventions, including tests, treatments, dietary changes, and prescription drugs. | There were significant improvements over time in news organizations’ success in meeting six of HNR’s 10 criteria for a successful health news story related to drugs, devices, surgery and other medical procedures, and diet; however, when data for television stories were excluded, only the improvement in avoiding disease-mongering remained significant. | Outcome |
| [20] | quantitative | secondary data | quantitative website content assessment | [We performed an environmental scan of currently available websites providing educational information about rheumatoid arthritis (RA) and evaluated the quality of these websites.](https://www.sciencedirect.com/topics/medicine-and-dentistry/rheumatoid-arthritis) | Current patient information on the Internet does not comprehensively address all educational needs of patients with RA, and is often outdated. | Current patient information on the Internet does not comprehensively address all educational needs of patients with RA, and is often outdated. The findings from our study highlight potential areas for improvement in online education materials for patients with RA. | Structure/Process |
| [21] | quantitative | primary data | observational registry-based study | It is our professional responsibility to share this information with them in a format they can understand. | [Three basic principles provide the rationale for the Society of Thoracic Surgeons (STS) Congenital Heart Surgery Database (CHSD) public reporting initiative: (1) Variation in congenital and pediatric cardiac surgical outcomes exist. (2) Patients and their families have the right to know the outcomes of the treatments that they will receive. (3).](https://www.sciencedirect.com/topics/medicine-and-dentistry/pediatrics) | [Therefore, the STS CHSD Mortality Risk Model provides excellent adjustment for case mix and should mitigate against risk aversive behavior. The STS CHSD Mortality Risk Model is the best available model to date for measuring outcomes after pediatric cardiac surgery.](https://www.sciencedirect.com/topics/medicine-and-dentistry/aversive-behavior) | Outcome |
| [22] | quantitative | primary data | Survey | We aim to identify factors that explain the (intended) use of HRC, including differences between current users and non-users. | Hospitals report cards (HRC) are publicly available Internet websites that provide patients with quality-related information on hospitals and enable hospital comparisons. | Health policy makers should focus on reviewing and restructuring the information content of HRC. In addition, coordinated efforts are still required to facilitate HRC access, especially for the ‘less fortunate’. | Structure |
| [23] | quantitative | primary data | data-driven analysis | Although individual quality measures provide important insight, it is challenging to understand hospital performance as characterized by multiple quality measures. | Public reporting of measures of hospital performance is an important component of quality improvement efforts in many countries. However, it can be challenging to provide an overall characterization of hospital performance because there are many measures of quality. | Our application of a novel graph analytics method to data describing U.S. hospitals revealed nuanced differences in performance that are obscured in existing hospital rating systems. | Process |
| [24] | qualitative | primary data | thematic content analysis | Our goal was to determine if tailoring quality reports to persons with diabetes mellitus and co-occurring chronic conditions would increase user engagement with a website that publicly reports the quality of diabetes care. | The majority of health care utilization decisions in the United States are made by persons with multiple chronic conditions. Existing public reports of health system quality do not distinguish care for these persons and are often not used by the consumers they aim to reach. | We conclude that tailoring can be used to improve public reporting sites for individuals with chronic conditions, ultimately allowing consumers to make more informed health care decisions. | Structure/Process |
| [25] | quantitative | primary data | cross-sectional observational study | We aimed to assess associations between publicly reported Medicare Advantage plan attributes (i.e., costs, quality, and benefits) and brand market share and beneficiaries’ enrollment decisions. | To facilitate informed decision-making in the Medicare Advantage marketplace, the Centers for Medicare & Medicaid Services publishes plan information on the Medicare Plan Finder website, including costs, benefits, and star ratings reflecting quality. Little is known about how beneficiaries weigh costs versus quality in enrollment decisions. | While beneficiaries prefer higher-quality and lower-cost Medicare Advantage plans, marginal utility for quality diminishes at higher star ratings, and their decisions are strongly associated with plans’ brand market share. | Structure |

**References:**

1. Sapin M, Ehlig D, Geissler A, Vogel J. Public reporting in five health care areas: A comparative content analysis across nine countries. Health Policy 2024 Dec 03;152:105222. doi: 10.1016/j.healthpol.2024.105222

2. Yilmaz NG, Timmermans DR, Van Weert JC, Damman OC. Breast cancer patients' visual attention to information in hospital report cards: An eye-tracking study on differences between younger and older female patients. Health Informatics Journal 2023 Jan 25;29(1). doi: 10.1177/14604582231155279

3. Temkin-Greener H, Mao Y, McGarry B. Online Customer Reviews of Assisted Living Communities: Association with Community, County, and State Factors. Journal of the American Medical Directors Association 2023 June;24(6):841-845.

4. Kast K, Otten S-M, Konopik J, Maier CB. Web-Based Public Reporting as a Decision-Making Tool for Consumers of Long-Term Care in the United States and the United Kingdom: Systematic Analysis of Report Cards. JMIR Formative Research 2023 Dec 14;7:e44382.

5. Gephart SM, Tolentino DA, Quinn MC, Wyles C. Neonatal intensive care workflow analysis informing NEC-zero clinical decision support design. CIN: Computers, Informatics, Nursing 2023 Feb;41(2):94-101. doi: 10.1097/CIN.0000000000000929

6. Shah NS, Umeda Y, Newyear B, Matar RN, Frederickson M, Parman MD, et al. Patients with higher postoperative pain after ambulatory shoulder surgery reported lower satisfaction: a prospective observational study. AME Surgical Journal 2022 Dec 01;2. doi: 10.21037/asj-22-11

7. Lopez-Olivo MA, des Bordes JK, Syed MN, Alemam A, Dodeja A, Abdel-Wahab N, et al. Quality appraisal of educational websites about osteoporosis and bone health. Archives of Osteoporosis 2021 Feb 10;16(1):28.

8. Lloyd S, Cliff C, FitzGerald G, Collie J. Can publicly reported data be used to understand performance in an Australian rural hospital? Health Information Management Journal 2021 Sep 16;50(1-2):35-46. doi: <https://doi.org/10.1177/1833358320948559>

9. Ivanković D, Barbazza E, Bos V, Brito Fernandes Ó, Jamieson Gilmore K, Jansen T, et al. Features constituting actionable COVID-19 dashboards: descriptive assessment and expert appraisal of 158 public web-based COVID-19 dashboards. Journal of Medical Internet Research 2021 Feb 24;23(2):e25682. doi: doi:10.2196/25682

10. Hennessee I, Clennon JA, Waller LA, Kitron U, Bryan JM. Considerations for improving reporting and analysis of date-based COVID-19 surveillance data by public health agencies. American Journal of Public Health 2021 Dec 8;111(12):2127-2132.

11. Blacketer C, Defalco FJ, Ryan PB, Rijnbeek PR. Increasing trust in real-world evidence through evaluation of observational data quality. Journal of the American Medical Informatics Association 2021 Jul 27;28(10):2251-2257. doi: <https://doi.org/10.1093/jamia/ocab132>

12. Barbazza E, Ivanković D, Wang S, Gilmore KJ, Poldrugovac M, Willmington C, et al. Exploring changes to the actionability of COVID-19 dashboards over the course of 2020 in the Canadian context: descriptive assessment and expert appraisal study. Journal of Medical Internet Research 2021 Aug 6;23(8):e30200. doi: 10.2196/30200

13. Barbazza E, Ivanković D, Davtyan K, Poldrugovac M, Yelgezekova Z, Willmington C, et al. The experiences of 33 national COVID-19 dashboard teams during the first year of the pandemic in the World Health Organization European Region: A qualitative study. Digital Health 2022 Aug 29;8. doi: <https://doi.org/10.1177/20552076221121154>

14. Ryskina KL, Andy AU, Manges KA, Foley KA, Werner RM, Merchant RM. Association of online consumer reviews of skilled nursing facilities with patient rehospitalization rates. JAMA network open 2020 May 14;3(5):e204682-e204682. doi: 10.1001/jamanetworkopen.2020.4682

15. Moore AR, Hudson C, Amey F, Chumbler N. Trusting sources of information on quality of physician care. INQUIRY: The Journal of Health Care Organization, Provision, and Financing 2020 Aug 24;57. doi: 10.1177/0046958020952912

16. Perraillon MC, Konetzka RT, He D, Werner RM. Consumer response to composite ratings of nursing home quality. American Journal of Health Economics 2019;5(2):165-190.

17. Hsu SH, Hung P, Wang S-Y. Factors Associated With Hospices' Nonparticipation in Medicare's Hospice Compare Public Reporting Program. Medical Care 2019 Jan;57(1):28-35. doi: 10.1097/MLR.0000000000001016

18. Cerully JL, Parker AM, Rybowski L, Schlesinger M, Shaller D, Grob R, et al. Improving patients’ choice of clinician by including roll-up measures in public Healthcare quality reports: an online experiment. Journal of General Internal Medicine 2018 Nov 16;34(2):243-249.

19. Walsh-Childers K, Braddock J, Rabaza C, Schwitzer G. One step forward, one step back: changes in news coverage of medical interventions. Health Communication 2018;33(2):174-187. doi: <https://doi.org/10.1080/10410236.2016.1250706>

20. Siddhanamatha HR, Heung E, de los Angeles Lopez-Olivo M, Abdel-Wahab N, Ojeda-Prias A, Willcockson I, et al. Quality assessment of websites providing educational content for patients with rheumatoid arthritis. Semin. Arthritis Rheum. 2017 May 20;46(6):715-723.

21. Jacobs JP. The society of thoracic surgeons congenital heart surgery database public reporting initiative. Seminars in Thoracic and Cardiovascular Surgery: Pediatric Cardiac Surgery Annual 2017 Jan;20:43-48. doi: <https://doi.org/10.1053/j.pcsu.2016.09.008>

22. Emmert M, Wiener M. What factors determine the intention to use hospital report cards? The perspectives of users and non-users. Patient Education and Counseling 2017 Jul;100(7):1394-1401.

23. Downing NS, Cloninger A, Venkatesh AK, Hsieh A, Drye EE, Coifman RR, et al. Describing the performance of US hospitals by applying big data analytics. PLoS One 2017 Jun 29;12(6):e0179603. doi: <https://doi.org/10.1371/journal.pone.0179603>

24. Smith MA, Bednarz L, Nordby PA, Fink J, Greenlee RT, Bolt D, et al. Increasing consumer engagement by tailoring a public reporting website on the quality of diabetes care: a qualitative study. Journal of Medical Internet Research 2016 Dec 21;18(12):e332. doi: doi:10.2196/jmir.6555

25. Reid RO, Deb P, Howell BL, Conway PH, Shrank WH. The roles of cost and quality information in Medicare Advantage plan enrollment decisions: an observational study. Journal of general internal medicine 2015 Aug 18;31:234-241.
