# Supplementary material for: Digital Public Reporting Systems for Evaluating Health Care Quality: Systematic Review
Source: JMIR Med Inform. 2026 Mar 18;14:e80435. doi: 10.2196/80435 (PMC12998539; doi:10.2196/80435)
Supplement: Multimedia Appendix 4 [file medinform-v14-e80435-s004.docx]

**Appendix 4. Details of the included studies**

| **Category (amounts of articles)** | **Main Findings** | **Source** |
| --- | --- | --- |
| Website presentation (n=10) | Standardized data guidelines help improve the quality of public reporting websites. | [1] |
|  | The format in which information is presented on public reporting websites can significantly influence public understanding and trust, policy-making, and responses to epidemics. | [2] |
|  | Two independent investigators evaluated the accuracy, completeness, technical elements, design and esthetics, readability, usability, and accessibility of the websites. | [3] |
|  | Quality, high-quality provider networks, and choice may be related; access to broad networks and preferred providers has been shown to influence plan selection. | [4] |
|  | online ratings may serve as a valuable source of information for both consumers and policymakers. | [5] |
|  | storytelling techniques that convey narrative trends on dashboards can help users better understand the information presented in public reporting. | [6] |
|  | supplementing with characteristics of professional care institutions, which are often underrepresented, can enhance the comprehensiveness of public reporting information. | [7] |
|  | the widespread use of aggregated ratings, such as star systems, may further strengthen consumer responses.. | [8] |
|  | Our results suggest that roll-ups in healthcare quality reports, alone or as a complement to drill-downs, can help patients make better decisions for themselves. | [9] |
|  | In addition, coordinated efforts are still required to facilitate HRC access, especially for the ‘less fortunate’. | [10] |
| Dashboard (n=5） | Clarity around data sources and indicator calculations (metadata) are critical for overall quality, credibility, and trustworthiness of reporting. For transparency on how data were collected and insights into “what lies behind” the reported indicators, providing explicit data sources and calculations should be considered a minimum requirement. | [11] |
|  | Improvements were most pronounced with regard to dashboard technology solutions (better customizable time trends, and new charts and graphs) and data provision (new indicators, more transparency on metadata, and more geographic granularity). Modifications to further develop communicative elements were less pronounced or even absent during the period assessed.. | [6] |
|  | Some saw the purpose as solely presenting data (raw numbers) for the public to interpret on its own; others endeavoured to provide explanations using narratives or visual methods. As reported in other studies, the ways in which information is presented may affect not only the subjective perception but also the objective comprehension of the information | [12] |
|  | Relevant and high-quality data are crucial for constructing dashboards, and research indicates that data sources are key factors in dashboard development. | [13] |
|  | the development of dashboards that allow users to create profiles and receive personalized scores enables consumers to “see themselves in the data”. | [14] |
| Data presentation (n=12) | Within the same country, websites using similar data sources may exhibit inconsistencies, which can create uncertainty for patients and their families regarding which hospital is the best choice. | [15] |
|  | Those should become more transparent and improve the reporting of prices and consumer feedback. Advanced search, simplification tools, and comparison functions should be integrated more widely. To make them more helpful for users and to bring public reporting a bit closer to its goal of improving the quality of health care services, both countries are advised to concentrate on optimizing the existing report cards. Those should become more transparent and improve the reporting of prices and consumer feedback. | [16] |
|  | Nurses described that “making a case” to the provider required them to describe symptoms within a context that could allow the provider to distinguish between different disease processes. However, some mentioned that they did not always know what was evolving but knew something “wasn't right” or that symptoms were not specific.  presenting data in a structured format on dashboards can facilitate clear communication between healthcare providers and patients. | [17] |
|  | Data across four domains, multiple measures and time periods were collected to examine access and equity; efficiency and sustainability; quality, safety and patient orientation; and employee engagement. | [13] |
|  | We describe the implementation of check types across a data quality framework of conformance, completeness, plausibility, with both verification and validation.  Transparently communicating how well common data model-standardized databases adhere to a set of quality measures adds a crucial piece that is currently missing from observational research. | [18] |
|  | We conclude that the form of quality reporting matters to consumers, and that the increased use of composite ratings is likely to increase consumer response. | [8] |
|  | Specifically, we built a semi-supervised machine learning algorithm and applied it to the publicly-available quality measures for 1,614 U.S. hospitals to graphically and quantitatively characterize hospital performance. In the resulting visualization, the varying density of hospitals demonstrates that there are key clusters of hospitals that share specific performance profiles, while there are other performance profiles that are rare. | [19] |
|  | The use of publicly reported data by consumers, policymakers, and researchers has grown substantially | [20] |
|  | Currently in the United States, the majority of health care utilization decisions are made by the quarter of individuals who have multiple chronic conditions, comprising approximately 66% of health care expenditures in the United States [38]. Having chronic conditions is a powerful motivator to seek out the best health care available because individuals with chronic conditions have a continuing need to know how to best manage their health conditions to avoid complications or deterioration, minimize symptoms, and improve their health [21]. | [14] |
|  | The format in which information is presented on public reporting websites can significantly influence public understanding and trust, policy-making, and responses to epidemics. | [2] |
|  | Public reporting websites can also serve as supplementary materials recommended by physicians, thereby reducing the need for face-to-face education and consultation | [3] |
|  | Standardized data guidelines help improve the quality of public reporting websites. | [1] |
| User Heterogeneity (n=4) | Publicly reported data can be used to represent performance for a rural hospital. Timeliness, level of detail available and peer groupings of data limits optimal utility. | [13] |
|  | Lessons emerged around the themes of simplicity, trust, partnership, software and data and change. | [12] |
|  | we found that within countries, same data used by different websites can lead to confusing or even contradictory information about the same provider, depending on the websites’ reporting methods and data usage. | [15] |
|  | In general, differences in level of health literacy, rather than (chronological) age, seem to be relevant to take into account when designing and/or updating HRCs. | [21] |
| Individual Characteristics and Contextual Factors (n=1) | Also, age was the only factor that significantly correlated with trusting information from all 3 sources. Specifically, younger adults trusted information from all sources compared to older adults. Furthermore, political affiliation, employment status, income, and area of residence correlated with trusting care quality information from either companies and government agencies or family and social network sources. Results suggest that individual and contextual characteristics are significant factors in trusting information sources regardless of health status and these should be taken into consideration by those promoting public reporting of healthcare quality information. | [22] |
| Patient satisfaction and surgical mortality (n=3) | Some studies have pointed out that the variables influencing patient risk levels in surgical mortality include demographic factors such as age, weight, and prematurity, surgical factors such as the type of procedure, as well as disease-related factors | [23] |
|  | Patient satisfaction is primarily influenced by pain levels at discharge and the accessibility of care, and is not associated with demographic characteristics (such as age or sex), surgical features, or preoperative function. The key determinants are: pain levels at discharge (the higher the pain, the lower the satisfaction) and the ability to obtain timely appointments. | [24] |
|  | In fact, for the latter criterion, there were statistically significant decreases in the percentage of satisfactory ratings for stories about drugs, tests, medical products, and diet, and the percentage of satisfactory ratings also declined, though not significantly, for stories about nondrug/nonsurgical treatments and procedures. | [25] |

**References:**

1. Lopez-Olivo MA, des Bordes JK, Syed MN, Alemam A, Dodeja A, Abdel-Wahab N, et al. Quality appraisal of educational websites about osteoporosis and bone health. Archives of Osteoporosis 2021 Feb 10;16(1):28. doi: 10.1007/s11657‑021‑00877‑x

2. Hennessee I, Clennon JA, Waller LA, Kitron U, Bryan JM. Considerations for improving reporting and analysis of date-based COVID-19 surveillance data by public health agencies. American Journal of Public Health 2021 Dec 8;111(12):2127-2132. doi: 10.2105/AJPH.2021.306520

3. Siddhanamatha HR, Heung E, de los Angeles Lopez-Olivo M, Abdel-Wahab N, Ojeda-Prias A, Willcockson I, et al. Quality assessment of websites providing educational content for patients with rheumatoid arthritis. Semin. Arthritis Rheum. 2017 May 20;46(6):715-723. doi: 10.1016/j.semarthrit.2017.01.006

4. Reid RO, Deb P, Howell BL, Conway PH, Shrank WH. The roles of cost and quality information in Medicare Advantage plan enrollment decisions: an observational study. J Gen Intern Med 2015 Aug 18;31:234-241. doi: 10.1007/s11606‑015‑3467‑3

5. Temkin-Greener H, Mao Y, McGarry B. Online customer reviews of assisted living communities: association with community, county, and state factors. Journal of the American Medical Directors Association 2023 June;24(6):841-845. doi: 10.1016/j.jamda.2023.02.007

6. Barbazza E, Ivanković D, Wang S, Gilmore KJ, Poldrugovac M, Willmington C, et al. Exploring changes to the actionability of COVID-19 dashboards over the course of 2020 in the Canadian context: descriptive assessment and expert appraisal study. J Med Internet Res 2021 Aug 6;23(8):e30200. doi: 10.2196/30200

7. Ryskina KL, Andy AU, Manges KA, Foley KA, Werner RM, Merchant RM. Association of online consumer reviews of skilled nursing facilities with patient rehospitalization rates. JAMA Netw Open 2020 May 14;3(5):e204682-e204682. doi: 10.1001/jamanetworkopen.2020.4682

8. Perraillon MC, Konetzka RT, He D, Werner RM. Consumer response to composite ratings of nursing home quality. American Journal of Health Economics 2019;5(2):165-190. doi: 10.1162/ajhe_a_00115

9. Cerully JL, Parker AM, Rybowski L, Schlesinger M, Shaller D, Grob R, et al. Improving patients’ choice of clinician by including roll-up measures in public Healthcare quality reports: an online experiment. J Gen Intern Med 2018 Nov 16;34(2):243-249. doi: 10.1007/s11606‑018‑4725‑y

10. Emmert M, Wiener M. What factors determine the intention to use hospital report cards? The perspectives of users and non-users. Patient Education and Counseling 2017 Jul;100(7):1394-1401. doi: 10.1016/j.pec.2017.01.021

11. Ivanković D, Barbazza E, Bos V, Brito Fernandes Ó, Jamieson Gilmore K, Jansen T, et al. Features constituting actionable COVID-19 dashboards: descriptive assessment and expert appraisal of 158 public web-based COVID-19 dashboards. J Med Internet Res 2021 Feb 24;23(2):e25682. doi: doi:10.2196/25682

12. Barbazza E, Ivanković D, Davtyan K, Poldrugovac M, Yelgezekova Z, Willmington C, et al. The experiences of 33 national COVID-19 dashboard teams during the first year of the pandemic in the World Health Organization European Region: A qualitative study. Digital Health 2022 Aug 29;8. doi: <https://doi.org/10.1177/20552076221121154>

13. Lloyd S, Cliff C, FitzGerald G, Collie J. Can publicly reported data be used to understand performance in an Australian rural hospital? Health Information Management Journal 2021 Sep 16;50(1-2):35-46. doi: <https://doi.org/10.1177/1833358320948559>

14. Smith MA, Bednarz L, Nordby PA, Fink J, Greenlee RT, Bolt D, et al. Increasing consumer engagement by tailoring a public reporting website on the quality of diabetes care: a qualitative study. J Med Internet Res 2016 Dec 21;18(12):e332. doi: doi:10.2196/jmir.6555

15. Sapin M, Ehlig D, Geissler A, Vogel J. Public reporting in five health care areas: A comparative content analysis across nine countries. Health Policy 2024 Dec 03;152:105222. doi: 10.1016/j.healthpol.2024.105222

16. Kast K, Otten S-M, Konopik J, Maier CB. Web-based public reporting as a decision-making tool for consumers of long-term care in the United States and the United Kingdom: systematic analysis of report cards. JMIR Form Res 2023 Dec 14;7:e44382. doi: 10.2196/44382

17. Gephart SM, Tolentino DA, Quinn MC, Wyles C. Neonatal intensive care workflow analysis informing NEC-zero clinical decision support design. Comput. Inform. Nurs. 2023 Feb;41(2):94-101. doi: 10.1097/CIN.0000000000000929

18. Blacketer C, Defalco FJ, Ryan PB, Rijnbeek PR. Increasing trust in real-world evidence through evaluation of observational data quality. J. Am. Med. Inform. Assoc. 2021 Jul 27;28(10):2251-2257. doi: <https://doi.org/10.1093/jamia/ocab132>

19. Downing NS, Cloninger A, Venkatesh AK, Hsieh A, Drye EE, Coifman RR, et al. Describing the performance of US hospitals by applying big data analytics. PLoS One 2017 Jun 29;12(6):e0179603. doi: <https://doi.org/10.1371/journal.pone.0179603>

20. Hsu SH, Hung P, Wang S-Y. Factors associated with hospices' nonparticipation in medicare's hospice compare public reporting program. Med. Care 2019 Jan;57(1):28-35. doi: 10.1097/MLR.0000000000001016

21. Yilmaz NG, Timmermans DR, Van Weert JC, Damman OC. Breast cancer patients' visual attention to information in hospital report cards: An eye-tracking study on differences between younger and older female patients. Health Informatics Journal 2023 Jan 25;29(1). doi: 10.1177/14604582231155279

22. Moore AR, Hudson C, Amey F, Chumbler N. Trusting sources of information on quality of physician care. INQUIRY: The Journal of Health Care Organization, Provision, and Financing 2020 Aug 24;57. doi: 10.1177/0046958020952912

23. Jacobs JP. The society of thoracic surgeons congenital heart surgery database public reporting initiative. Seminars in Thoracic and Cardiovascular Surgery: Pediatric Cardiac Surgery Annual 2017 Jan;20:43-48. doi: <https://doi.org/10.1053/j.pcsu.2016.09.008>

24. Shah NS, Umeda Y, Newyear B, Matar RN, Frederickson M, Parman MD, et al. Patients with higher postoperative pain after ambulatory shoulder surgery reported lower satisfaction: a prospective observational study. AME Surgical Journal 2022 Dec 01;2. doi: 10.21037/asj-22-11

25. Walsh-Childers K, Braddock J, Rabaza C, Schwitzer G. One step forward, one step back: changes in news coverage of medical interventions. Health Communication 2018;33(2):174-187. doi: <https://doi.org/10.1080/10410236.2016.1250706>
